# Head-to-Head Comparison of CZT-SPECT and SPECT/CT Myocardial Perfusion Imaging: Interobserver and Intraobserver Agreement and Diagnostic Performance

**DOI:** 10.3390/life13091879

**Published:** 2023-09-07

**Authors:** Forough Kalantari, Nasibeh Mohseninia, Andreas Wetsch, Sara Harsini, Lukas Hehenwarter, Gregor Schweighofer-Zwink, Nazanin Zamani-Siahkali, Gundula Rendl, Mohsen Beheshti, Christian Pirich

**Affiliations:** 1 Division of Molecular Imaging and Theranostics, Department of Nuclear Medicine, University Hospital, Paracelsus Medical University, 5020 Salzburg, Austrian.zamani@salk.at (N.Z.-S.);; 2 BC Cancer Research Institute, Vancouver, BC V5Z 1L3, Canada; 3 Research Center for Nuclear Medicine, Tehran University of Medical Sciences, Tehran 1411713135, Iran

**Keywords:** SPECT, reproducibility, coronary artery disease, myocardial perfusion imaging

## Abstract

Background: Myocardial perfusion imaging (MPI) plays a crucial role in diagnosing coronary artery disease (CAD), with single-photon emission computed tomography (SPECT) being a widely accepted method. The accuracy of MPI relies on image quality and the expertise of physicians. While CZT-SPECT cameras offer advantages, they can be susceptible to attenuation artifacts. Therefore, our objective was to evaluate the diagnostic accuracy of CZT-SPECT and SPECT/CT in a clinical setting. Method: We conducted a prospective single-center study involving patients with known or suspected stable ischemic heart disease who underwent SPECT-MPI using CZT-SPECT and SPECT/CT scanners, and the latter was equipped with cardiofocal collimation. Experienced physicians performed analysis and reporting based on automated quantification and visual image interpretation. Results: A total of 77 patients (32 women (41.6%) and 45 men (58.4%) with an average age of 71.9 ± 8.9 years) were included. The agreement between readers regarding the final conclusion based on imaging reporting using both devices was very high (Kappa 0.87–0.93). Per-vessel analysis revealed a trend suggesting that CZT-SPECT was superior to conventional SPECT/CT in terms of sensitivity, positive predictive value (PPV), negative predictive value (NPV), and accuracy, although the difference did not reach statistical significance. Conclusion: Our study demonstrated that CZT-SPECT imaging offers comparable diagnostic accuracy, improved patient comfort, and eliminates CT-induced radiation compared to SPECT/CT. These findings suggest that cardiac CZT-SPECT imaging has the potential to become a valuable imaging modality in clinical practice.

## 1. Introduction

Coronary artery disease (CAD) is the leading cause of death worldwide and a significant public health challenge among both genders [1,2]. Myocardial perfusion imaging (MPI) using single-photon emission computed tomography (SPECT) is a non-invasive diagnostic method for assessing CAD [3]. It is widely accepted as a powerful gatekeeper for cardiac catheterization, as it determines the functional and prognostic relevance of CAD [4,5]. It is essential to optimize non-invasive diagnostic imaging for patients with suspected CAD, as a significant proportion of invasive coronary angiograms (ICA) fail to demonstrate any evidence of obstructive disease. Up to two-thirds of cases show no such evidence [6].

In general, MPI reporting is based on the visual assessment of fractional tracer uptake in different myocardial segments of the left ventricle, taking into account the pretest probability of disease, image quality, and potential artifacts [4]. According to current guidelines, automated quantitative analyses should also be used as complementary data to visual interpretation [4]. However, it is important to note that most recommendations are primarily derived from conventional SPECT-MPI technology [4]. Although SPECT-MPI has been routinely performed for more than 20 years, SPECT hardware and image processing software are still evolving. This requires consistent customization of acquisition and reconstruction parameters to improve its diagnostic accuracy, shorten acquisition times, and reduce radiation exposure [7,8,9].

MPI is susceptible to interference from various types of artifacts, including patient-related artifacts, equipment-related artifacts, and technical-associated etiologies [10]. One of the most common artifacts in MPI is caused by the attenuation of photons by the patient’s body [11]. Computed tomography-attenuation correction (CT-AC) is a validated approach to improve diagnostic accuracy and risk stratification [12]. According to the American Society of Nuclear Cardiology’s recommendation, attenuation correction should be performed in all patients [13]. A commonly used and widely accepted scanner type for MPI is standard dual-head hybrid SPECT/computed tomography (CT). It has the advantages of attenuation correction, integration of calcium score, and CT-based morphological co-registration of the coronary arteries. The integration of perfusion imaging with morphological findings and coronary calcium scoring provides a unified approach to assessing functional and morphological abnormalities in a single setting [9]. 

Another main technological advance in SPECT cameras is high-efficiency scanners incorporating cadmium zinc telluride (CZT) solid-state detectors (SSDs) [14]. CZT detectors have also been introduced for dedicated cardiac SPECT systems in clinical practice [15]. The new systems have improved count sensitivity and image quality [14]. The use of CZT-SPECT in clinical practice is on the rise due to its benefits of enhanced image accuracy and reduced scanning duration compared to conventional SPECT systems using sodium iodide (NaI) crystals [12].

This article presents prospective data of head-to-head comparison of MPI findings in a cohort examined by both CZT-SPECT (D-SPECT, Spectrum Dynamics Medical Ltd., Caesarea, Israel) and SPECT/CT (Symbia T6, Siemens healthineers, Erlangen, Germany). 

## 2. Materials and Methods

### 2.1. Study Population

This prospective single-center study was performed in accordance with the principles of the 1964 Declaration of Helsinki and its later amendments or comparable standards and approved by the ethics committee of the province with the trial number of “EK-1030/2019”. Overall, 77 patients consisted of 32 women (41.6%) and 45 men (58.4%) with known or suspected stable ischemic heart disease. The median age of the study cohort was 71.9 ± 8.9 years, with a range of 38 to 86 years. As a routine protocol in our department, all patients were generally considered for the one-day protocol, except for inpatients and/or those who were unable to comply with the one-day protocol. Sixteen of the patients underwent contrast coronary angiography within one month after the SPECT MPI. Pressure wire and functional analysis were performed if required (*n* = 1). Six patients underwent angioplasty following the abnormal angiography. Qualitatively suboptimal scintigraphy, which was affected by artifacts, was excluded from the study. Signature of the written informed consent was obtained from all individual participants included in the study according to the guidelines of the Ethics Committee.

### 2.2. Stress and Acquisition Protocols

All patients were examined with SPECT-MPI using technetium (^99m^Tc) tetrofosmin on both scanners consequently: CZT-SPECT (D-SPECT, Spectrum Dynamics Medical Ltd., Caesarea, Israel) and SPECT/CT (Symbia T6, Siemens healthineers, Erlangen, Germany) equipped with cardiofocal collimation. The pharmacologic stress method used to induce coronary dilatation before stress imaging for all patients was the regadenoson-only method. A dose of 0.4 mg/5 mL of regadenoson was administered into a peripheral vein using a 22-gauge or larger catheter or needle, immediately followed by a 5 mL flush of saline. The injected activity was 2.5–3.5 MBq/kg/body-weight (bw) for stress and 7.5–10.5 MBq/kg/bw for rest. Following the ALARA principles and to avoid additional radiation exposure, stress-only MPI was performed in all patients who showed normal stress MPI on both CZT-SPECT and SPECT/CT cameras. Out of the 77 patients, 54 underwent the stress-only protocol, while 23 were examined using the stress and rest protocol. Among the patients who had the stress and rest protocol, 14 underwent a two-day protocol, and nine underwent a one-day stress–rest protocol. The mean doses of ^99m^Tc-tetrofosmin administered for stress and rest imaging were 334.8 ± 50.1 MBq (range 252–459) and 577.8 ± 176.7 MBq (range 282–838), respectively. The median time between CZT-SPECT and Symbia-SPECT/CT stress studies was 17 min (range 7–72 min.). The radiotracer was administrated within 10 to 20 s using the same IV line, followed by a 5 mL saline flush, and stress images were acquired 45 min later.

### 2.3. Automated Quantification

Automated quantification and quality control were performed by one experienced technologist who was blinded to clinical data. In case of need, myocardium contour correction was also applied. Quantitative perfusion and quantitative gated SPECT functional results were obtained using site-specific quantification software (automated 4DM software). The polar map of the left ventricular myocardium was divided into 17 segments. The radiotracer uptake in each myocardium segment was scored on a scale of zero to four, with zero representing maximum uptake and four representing no uptake (zero = normal, one = mildly decreased, two = moderately decreased, three = severely decreased, and four = absence of uptake). The summation of the scores for all segments in stress and rest represents the summed stress score (SSS) and summed rest score (SRS), respectively. The SSS, SRS, summed difference score (SDS), stress total blackout, and rest total blackout were documented. ECG-gated functional information was extracted, including, ejection fraction (EF), end diastolic volume (EDV), end systolic volume (ESV), summed thickness score (STS), and summed motion score (SMS). 

### 2.4. Visual Interpretation

All CZT-SPECT and Symbia-SPECT/CT images were reviewed and interpreted by two experienced board-certified nuclear medicine physicians who were blinded to the clinical data and the results of the coronary angiography. The interpretation was based on visual segmental perfusion abnormalities. Patient management and angiography were performed based on the final clinical report from the responsible physician.

The left ventricular myocardial segments were assessed on a five-point scale, with scores ranging from zero (indicating normal perfusion) to four (indicating no perfusion). A score of one indicated minimal/mild or ambiguous perfusion defects, while a score of two represented moderate defects, and a score of three indicated severe defects. 

The SSS was classified into four groups: normal (SSS = 0), probably normal (SSS = 1), equivocal (SSS = 2–3), or abnormal (SSS ≥ 4). The coronary angiography scoring system used a scale of zero to three, with zero indicating normal studies, one indicating one-vessel disease, two indicating two-vessel disease, and three indicating three-vessel disease. The imaging times for both scanners ranged from 4 to 6 min [16].

### 2.5. Statistical Analysis

Descriptive statistics were expressed as the mean (±SD) or median (range) if the data for a given variable were not normally distributed according to the Kolmogorov–Smirnov test. The paired t-test was performed to compare differences in normal variables between the Symbia-SPECT/CT and CZT-SPECT studies; otherwise, nonparametric tests, including the Wilcoxon signed-rank test for paired data, were used. To adjust for multiple testing and control the false discovery rate (FDR), the Benjamini–Hochberg method was used [17]. Agreement within and between observers (readers 1 and 2) was calculated using the Cohen kappa or Weighted kappa coefficients for categorical variables with two levels or more than two levels, respectively. Agreement for continuous numerical data was measured using the two-way intraclass correlation coefficient (ICC). The ICC and kappa values indicated no agreement if they were < 0; slight agreement if they were 0.00–0.20; fair agreement if they were 0.21–0.40; moderate agreement if they were 0.41–0.60; substantial agreement if they were 0.61–0.80; and almost perfect agreement if they were 0.81–1.00. Sensitivity, specificity, positive predictive value (PPV), negative predictive value (NPV), and accuracy were calculated for each reader and method for detecting obstructive lesions (≥50% luminal narrowing according to invasive coronary angiography) using standard formulas. The McNemar test was used to compare the performance of the two diagnostic modalities for detecting obstructive lesions. A Chi-square test of independence was conducted to evaluate the association between the camera and attenuation location. Statistical analyses were conducted using IBM SPSS Statistics 28.0 (IBM Corporation, Armonk, NY, USA) and R (version 4.1.2; The R Foundation for Statistical Computing, General Public License). All tests were two sided, and a *p* value of less than 0.05 was considered significant.

## 3. Results

Table 1 presents the baseline characteristics of the patients included in the study. Of the 16 patients who underwent invasive coronary angiography following myocardial perfusion imaging, 12 (75.0%) had angiographically significant disease defined as stenosis above 50%. According to the results of invasive coronary angiography, four (25.0%) had no significant stenosis, four (25.0%) had single-vessel disease, two (12.5%) had two-vessel disease, and six (37.5%) had three-vessel disease.

Table 2 presents the intraobserver agreement of the readers. Almost perfect agreement was observed with reader 2 for the final conclusion, and with reader 1 for visual SSS. The reproducibility of final conclusion, presence of scar, and visual SDS was found to be substantial with reader 1, and for the presence of scar, visual SSS, and visual SDS with reader 2. Moderate concordance and reproducibility were observed with both readers for the presence, territory, and severity of ischemia, as well as TID with reader 2. Fair concordance was observed with both readers for the presence of attenuation, attenuation location, and artifact severity, and with reader 1 for the presence of artifact and gastrointestinal artifact. Slight agreement was noted for TID with reader 1, and for the presence of artifact and gastrointestinal artifact with reader 2 (Table 3).

The data regarding interobserver agreement are presented in Table 4. The agreement between the two readers was almost perfect for the final conclusion in both Symbia-SPECT/CT and CZT-SPECT categories, as well as for the presence and severity of ischemia and visual SSS in Symbia-SPECT/CT and TID in CZT-SPECT. Substantial agreement was observed for the territory of ischemia, presence of attenuation, attenuation location, scar, and visual SDS for both CZT-SPECT and Symbia-SPECT/CT, artifact severity and TID in Symbia-SPECT/CT, and presence and severity of ischemia and visual SSS for CZT-SPECT. The levels of agreement for the presence of artifact in both CZT-SPECT and Symbia-SPECT/CT, artifact severity in CZT-SPECT, and gastrointestinal artifact in Symbia-SPECT/CT were moderate. The lowest concordance was observed for gastrointestinal artifact in CZT-SPECT.

CZT-SPECT and Symbia-SPECT/CT image-derived parameters and their comparison on the basis of the camera model in the subgroup of patients who subsequently underwent invasive coronary angiography (*n* = 16) are presented in Table 5. As shown, the differences were statistically significant only for the stress EF gated (*p* = 0.031), stress SMS gated (*p* = 0.031), rest ESV gated (*p* = 0.011), and rest EF gated (*p* = 0.011). However, there was a significant difference between most quantitative parameters when compared across the whole cohort. (Table 6).

**Figure 1:**
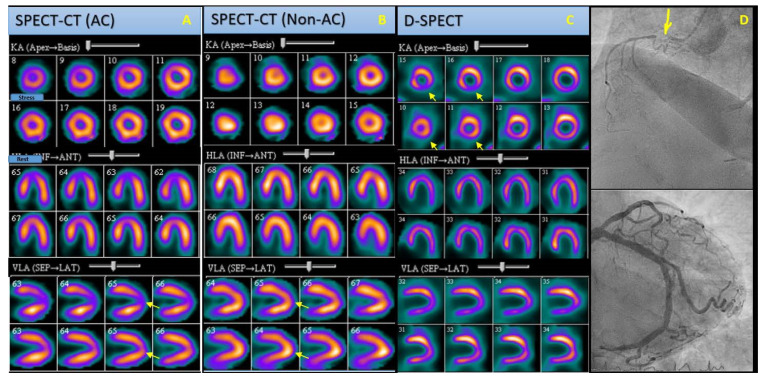
SPECT-CT and CZT-SPECT cardiac perfusion (stress: upper rows, rest: lower rows). Polar maps and angiography images of an obese 82-year-old woman with clinical presentation of stable angina and a medical history of hypertension. (**A**) Non-AC SPECT-CT images reveal reduced radiotracer uptake in the apical wall during the stress phase, which is reversed on rest phase images (arrows), indicating either an attenuation artifact or a reversible perfusion defect. (**B**) AC SPECT-CT images demonstrating a normal perfusion scan (arrows). (**C**) CZT-SPECT images present reduced activity in the inferior wall in stress phase, which is reperfused on rest phase images (arrows), indicative of reversible ischemia in the inferior wall. (**D**) Angiography images of the same patient demonstrating presence of stenosis in RCA (arrow), which correlates perfectly with the CZT-SPECT results. *SPECT-CT: Single Photon Emission Computed Tomography-Computed Tomography; CZT-SPECT: Digital Single Photon Emission Computed Tomography; Non-AC: non-Attenuation Corrected; AC: Attenuation Corrected, RSA: Right Coronary Artery*.

Using a per-vessel analysis, CZT-SPECT (reader 1) demonstrated a sensitivity of 81%, specificity of 64%, PPV of 72%, NPV of 74%, and accuracy of 73%. In comparison, imaging with Symbia-SPECT/CT (reader 1) showed a sensitivity of 69%, specificity of 64%, PPV of 69%, and NPV of 64%, and accuracy of 67%. For reader 2, sensitivity, specificity, PPV, NPV, and accuracy were 81%, 48%, 67%, 67%, and 0.67% with CZT-SPECT and 73%, 45%, 61%, 59%, and 60% with Symbia-SPECT/CT (Table 7). Per-vessel, CZT-SPECT demonstrated a trend toward higher accuracy than imaging with Symbia-SPECT/CT (73% versus 67% for reader 1; 67% versus 60% for reader 2), although the difference did not reach statistical significance (Figure 1).

The per-patient diagnostic performance of both imaging modalities, using both readers’ interpretation and parameters derived from scans, in comparison with invasive coronary angiography is shown in Table 8. The CZT-SPECT images showed a per-patient sensitivity, specificity, PPV, NPV, and accuracy of 82%, 50%, 90%, 33%, and 77%, respectively, for predicting CAD in invasive coronary angiography. In comparison, Symbia-SPECT/CT images showed a sensitivity of 54%, specificity of 0%, PPV of 75%, NPV of 0%, and accuracy of 46% for predicting CAD in invasive coronary angiography. We noted a statistically non-significant trend towards improved sensitivity, specificity, PPV, NPV, and accuracy for detection of significant coronary stenosis with CZT-SPECT compared to imaging with Symbia-SPECT/CT.

## 4. Discussion

In this study, we evaluated by direct comparison the diagnostic performance of the CZT-SPECT and collimation systems in the CZT-SPECT system, in comparison with the computed tomography attenuation correction-SPECT system with cardiofocal collimation, in a clinical setting for the detection of myocardial ischemia. While several studies have primarily focused on the superior aspects of the CZT-SPECT system in their reports [15,18,19], there are limited data on head-to-head performance analysis in routine clinical practice.

In medical imaging, the interpretation of results can be subjective and influenced by technical factors [20]. Therefore, it is crucial to optimize and maintain consistent parameters to ensure reliable and meaningful results [20]. To mitigate potential bias from subjective interpretation and inter-reader variability, we assessed both intraobserver and interobserver agreement. We found a high level of agreement between the readers, indicating consensus on the final conclusion, scar detection, and visual SSS, regardless of the device used. The interobserver agreement using both CZT-SPECT and Symbia-SPECT/CT technologies was almost perfect in determining the final conclusion and the presence of ischemia. 

Automated analysis of myocardial perfusion has become a routine part of daily practice in nuclear cardiology as it can enhance visual assessment and offer high reproducibility [21]. Our data aligns with previous studies, indicating the reproducibility of quantitative analysis, and we found no significant difference between the quantitative parameters derived from CZT-SPECT and SPECT/CT, including EDV, STS, SSS, SRS, SDS, and total blackout score. A phantom-based study comparing CZT-SPECT images with conventional cameras showed lower SDS with CZT-SPECT, and the use of attenuation correction reduced this difference [22]. However, we did not find any significant difference between the SDS values of the two cameras.

We found an exception with a statistically significant difference when comparing the quantitative parameters derived from CZT-SPECT and SPECT/CT. There was a statistically significant difference between stress and rest EF, rest ESV, and stress SMS. Although the difference was not statistically significant, the trend of stress ESV was consistent with that of rest ESV. Since the EDV was constant, the lower EF can be justified by a lower ESV in CZT-SPECT. This finding aligns with the higher spatial resolution of the CZT-SPECT camera compared to conventional SPECT cameras [15,23]. It can also explain our almost excellent agreement for TID detection and substantial agreement of scar detection in CZT-SPECT and SPECT/CT, with a non-significantly higher trend on CZT-SPECT. 

The results of our study revealed a trend toward better diagnostic performance and higher per-vessel diagnostic accuracy of CAD detection with CZT-SPECT compared to SPECT/CT imaging. We observed a greater number of false-negative results on SPECT/CT than on CZT-SPECT, leading to the higher sensitivity and accuracy of CZT-SPECT. One possible explanation is that the higher sensitivity and resolution of CZT-SPECT may enable the detection of smaller and less severe abnormalities. Our result are consistent with previously published studies [15,24,25], which highlight the superior energy resolution, detector sensitivity, and spatial resolution of CZT-SPECT compared to anger SPECT studies. These studies demonstrated improved per-vessel detection of CAD and significant enhancement in the delineation of multivessel CAD compared to standard SPECT/CT. The increased sensitivity of CZT-SPECT may also help reduce attenuation artifacts and ultimately contribute to higher diagnostic accuracy [24].

We did not observe a superior trend of CZT-SPECT in terms of specificity. However, previous reports with larger patient cohorts have a wide range of specificity from 37% to 93% [26]. Speculation suggests that the lower specificity of CZT-SPECT imaging may be attributed to the practice of performing upright imaging for patient comfort, which can lead to increased abdominal attenuation artifacts. Additionally, its use in the obese patient population is associated with a higher number of false positives [12]. In our study, the higher proportion of males to females (87.5%) and the presence of obesity (average BMI 31 ± 5.4) in the subgroup who underwent angiography may explain this result. Therefore, it is important to consider the difference in location and extent of attenuation artifacts when using a CZT-SPECT camera to avoid misinterpretation [27].

The interobserver agreement for the presence of artifacts in both Symbia-SPECT/CT and CZT-SPECT, the severity of artifacts in CZT-SPECT, and gastrointestinal artifact in SPECT/CT was moderate, while the intraobserver agreement for these parameters was fair. This may be partly attributed to two main factors: the time interval between the two series of imaging and the physiologic movements of gastrointestinal activity, as well as differences in patient positioning. 

The occurrence rate of soft tissue attenuation in SPECT MPI scans is estimated to be between 17% to 49%, making it a common artifact, especially in obese patients [28]. Our study revealed similar results, as we found that the incidence of attenuation artifacts was less than 30%, with no statistically significant difference between the two devices and two readers. This finding was expected since the overall BMI of our patients was in the overweight category, with an average BMI of 27.7 ± 5.6. Moreover, the presence of extracardiac soft tissue can degrade the performance of CZT-SPECT cameras, and attenuation correction methods are still needed [22].

We are aware of the limitations of the study, including the low number of gold standard angiographies and unavailable on-site catheterization data. Subgrouping the data resulted in a significant reduction in the number of individuals in each group, which reduced the statistical power and led to a disproportion of males to females with an average BMI > 30 (obesity) in the subgroup that underwent angiography. However, our study’s strength lies in comparing both quantitatively derived data and interobserver and intraobserver agreement in clinical routine practice. Additionally, there was no directional patient selection bias as the study was conducted on a general population of patients referred to our center in routine practice.

## 5. Conclusions

Our study found a trend toward higher per-patient and per-vessel accuracy with CZT-SPECT compared to SPECT/CT imaging. It offers the benefit of a single comfortable patient position and eliminates the radiation induced by computed tomography, while providing a diagnostic accuracy level comparable to SPECT/CT, with no significant difference in confounding attenuation. This new generation of CZT-SPECT gamma cameras, such as CZT-SPECT imaging, can offer excellent image quality scores and at least the same, if not better, performance in clinical practice as SPECT/CT.

## Figures and Tables

**Table 1:** Baseline characteristics.

*n* = 77	
Gender, male (*n*, %)	45 (58.4%)
Age, years (mean ± SD)	71.9 ± 8.9
Weight, kg (mean ± SD)	82.0 ± 15.6
Height, cm (mean ± SD)	170.8 ± 8.5
BMI (mean ± SD) Patients with known CAD (*n*) Patients with HTN (*n*) Patients with DM (*n*) Patients with dyslipidemia (*n*)	27.7 ± 5.6 27 53 16 41
Injected activity at stress test, MBq (mean ± SD)	334.8 ± 50.1
Injected activity at rest test ^§^, MBq (mean ± SD)	577.8 ± 176.7
Time interval between the two acquisitions for stress test ^£^, mins (median (range))	17 (7–72)

^§^ *n* = 22. ^£^ In all cases, CZT-SPECT imaging was performed first, followed by imaging with Symbia-SPECT/CT camera. CAD, coronary artery disease; HTN, arterial hypertension; DM, diabetes mellitus.

**Table 2:** Intraobserver agreement (*n* = 77).

Parameter	Reader 1		Reader 2	
	Kappa (95% CI)	Weighted Kappa	Kappa (95% CI)	Weighted Kappa
Final conclusion	0.798 (0.643 to 0.952)	-	0.811 (0.668 to 0.953)	-
Perfusion defect/ischemia	0.560 (0.383 to 0.737)	-	0.564 (0.396 to 0.733)	-
Territory	0.344 (0.219 to 0.470)	0.453	0.350 (0.229 to 0.470)	0.507
Severity	0.402 (0.256 to 0.547)	0.568	0.354 (0.216 to 0.492)	0.532
Attenuation	0.270 (0.049 to 0.492)	-	0.247 (0.024 to 0.471)	-
Attenuation location	0.225 (0.055 to 0.395)	0.229	0.249 (0.067 to 0.430)	0.223
Presence of artifact	0.279 (0.064 to 0.494)	-	0.113 (−0.144 to 0.369)	-
Artifact severity	0.172 (0.035 to 0.309)	0.273	0.120 (−0.016 to 0.257)	0.223
Gastrointestinal artifact	0.323 (0.114 to 0.532)	-	0.095 (−0.154 to 0.344)	-
Scar	0.762 (0.584 to 0.960)	-	0.725 (0.499 to 0.952)	-
TID	0.147 (−0.197 to 0.491)	-	0.529 (0.197 to 0.860)	-
	**ICC (95% CI)**		**ICC (95% CI)**	
Visual SSS	0.822 (0.734 to 0.883)		0.719 (0.591 to 0.812)	
Visual SDS	0.788 (0.813 to 0.924)		0.672 (0.529 to 0.779)	

95% CI, 95% confidence intervals; SSS, summed stress score; SDS, summed difference score; TID, transient ischemic dilatation; ICC, intraclass correlation coefficient.

**Table 3:** Attenuation location in Symbia-SPECT/CT and CZT-SPECT studies (*n* = 77).

Attenuation	Reader 1			Reader 2		
	SPECT/CT	CZT-SPECT	*p* Value ^†^	SPECT/CT	CZT-SPECT	*p* Value ^†^
None	49 (63.6%)	50 (64.9%)	0.97	53 (68.8%)	49 (63.6%)	0.67
Breast attenuation	12 (15.6%)	11 (14.3%)		12 (15.6%)	10 (13.0%)	
Diaphragmatic attenuation	12 (15.6%)	13 (16.9%)		9 (11.7%)	14 (18.2%)	
Breast + diaphragmatic attenuation	4 (5.2%)	3 (3.9%)		3 (3.9%)	4 (5.2%)	

^†^ *p* values from Chi-square test.

**Table 4:** Interobserver agreement (*n* = 77).

Parameter	SPECT/CT		CZT-SPECT	
	Kappa (95% CI)	Weighted Kappa	Kappa (95% CI)	Weighted Kappa
Final conclusion	0.930 (0.835 to 1.000)	-	0.876 (0.759 to 0.993)	-
Perfusion defect/ischemia	0.974 (0.923 to 1.000)	-	0.799 (0.658 to 0.940)	-
Territory	0.755 (0.646 to 0.865)	0.777	0.750 (0.639 to 0.862)	0.781
Severity	0.779 (0.666 to 0.891)	0.850	0.609 (0.473 to 0.745)	0.708
Attenuation	0.711 (0.545 to 0.876)	-	0.663 (0.489 to 0.837)	-
Attenuation location	0.699 (0.546 to 0.852)	0.704	0.685 (0.530 to 0.840)	0.679
Presence of artifact	0.467 (0.256 to 0.687)	-	0.444 (0.264 to 0.625)	-
Artifact severity	0.522 (0.387 to 0.657)	0.685	0.508 (0.370 to 0.647)	0.567
Gastrointestinal artifact	0.446 (0.237 to 0.654)	-	0.383 (0.203 to 0.562)	-
Scar	0.656 (0.401 to 0.910)	-	0.723 (0.514 to 0.931)	-
TID	0.708 (0.398 to 1.000)	-	0.916 (0.753 to 1.000)	-
	**ICC (95% CI)**		**ICC (95% CI)**	
Visual SSS	0.904 (0.853 to 0.938)		0.782 (0.677 to 0.856)	
Visual SDS	0.758 (0.644 to 0.839)		0.639 (0.486 to 0.755)	

95% CI, 95% confidence intervals; SSS, summed stress score; SDS, summed difference score; TID, transient ischemic dilatation; ICC, intraclass correlation coefficient.

**Table 5:** Comparison of Symbia-SPECT/CT and CZT-SPECT studies in patients followed with invasive coronary angiography (*n* = 16).

Parameter	Stress Test			Rest Test		
	SPECT/CT (*n* = 16)	CZT-SPECT (*n* = 16)	Adj. *p* Value ^Ψ^	SPECT/CT (*n* = 13)	CZT-SPECT (*n* = 13)	Adj. *p* Value ^Ψ^
EDV gated	147.81 ± 60.46	136.37 ± 33.84	0.300	149.54 ± 45.90	145.38 ± 48.02	0.467
ESV gated	81.94 ± 62.80	60.00 ± 28.60	0.097	**86.61 ± 48.69**	**70.69 ± 52.03**	**0.011**
EF gated	**49.19 ± 15.74**	**57.56 ± 12.07**	**0.031**	**45.08 ± 15.10**	**55.38 ± 18.06**	**0.011**
STS gated	7 (1–31)	11.5 (3–34)	0.097	7 (1–32)	18 (0–43)	0.121
SMS gated	**15.5 (0–58)**	**5.5 (0–46)**	**0.031**	13 (1–49)	5 (0–51)	0.374
SSS NC/SA	9 (0–22)	11.5 (2–24)	0.257	-	-	
SRS NC/SA	-	-		4 (0–19)	9 (0–20)	0.647
SDS ^£^ NC/SA	-	-		7 (0–13)	7 (0–10)	0.936
Total blackout score NC	19.5 (0–43)	21 (0–38)	0.756	5 (0–32)	15 (0–43)	0.448
SSS SC/gated	12.5 (0–30)	8 (0–17)	0.081	-	-	
SRS SC/gated	-	-		5 (0–19)	5 (0–19)	0.433
SDS ^£^ SC/gated	-	-		5 (0–18)	6 (1–11)	0.791
Total blackout score SC/gated	23 (0–70)	17 (0–34)	0.081	11 (0–55)	12 (0–39)	0.448

EDV, end-diastolic volume; ESV, end-systolic volume; EF, ejection fraction; NC, non-corrected; SC, scatter corrected; STS, summed thickening score; SMS, summed motion score; SSS, summed stress score; SRS, summed rest score; SDS, summed difference score. Values are presented as mean ± standard deviation and median (range) for normally distributed and non-normally distributed data, respectively. The bold values indicate statistical significance at the α = 0.05 level. ^Ψ^ Adjusted *p*-value for multiple testing using Benjamini–Hochberg method. ^£^ Assessed in patients with both stress and rest tests (*n* = 13).

**Table 6:** Comparison of Symbia and CZT-SPECT studies in the entire cohort (*n* = 77).

Parameter	Stress Test			Rest Test		
	SPECT/CT (*n* = 77)	CZT-SPECT (*n* = 77)	Adj. *p* Value ^Ψ^	SPECT/CT (*n* = 23)	CZT-SPECT (*n* = 23)	Adj. *p* Value ^Ψ^
EDV gated	**114.44 ± 47.17**	**108.65 ± 38.68**	**0.026**	139.68 ± 42.03	138.73 ± 41.44	0.803
ESV gated	**54.65 ± 39.93**	**42.75 ± 28.85**	**0.0001**	**77.59 ± 40.69**	**65.14 ± 41.41**	**0.001**
EF gated	**56.04 ± 13.48**	**63.53 ± 11.95**	**0.0001**	**46.77 ± 13.55**	**55.55 ± 15.11**	**<0.001**
STS gated	**4 (0–41)**	**15 (0–48)**	**<0.0001**	**5.5 (0–38)**	**17 (0–51)**	**0.008**
SMS gated	**8 (0–58)**	**2 (0–46)**	**<0.0001**	**13.5 (1–49)**	**6.5 (0–51)**	**0.009**
SSS NC/SA	2 (0–28)	3 (0–24)	0.270	-	-	
SRS NC/SA	-	-		4.5 (0–25)	7 (0–20)	0.463
SDS ^£^ NC/SA	-	-		4.5 (0–13)	4.5 (0–11)	0.539
Total blackout score NC	3 (0–69)	5 (0–43)	0.200	8.5 (0–53)	15 (0–43)	0.289
SSS SC/gated	**3 (0–39)**	**1 (0–17)**	**0.015**	-	-	
SRS SC/gated	-	-		5 (0–19)	4 (0–19)	0.463
SDS ^£^ SC/gated	-	-		3 (0–18)	4.5 (0–13)	0.968
Total blackout score SC/gated	**3 (0–85)**	**2 (0–34)**	**0.040**	9 (0–55)	8 (0–39)	0.463

EDV, end-diastolic volume; ESV, end-systolic volume; EF, ejection fraction; NC, non-corrected; SC, scatter corrected; STS, summed thickening score; SMS, summed motion score; SSS, summed stress score; SRS, summed rest score; SDS, summed difference score. Values are presented as mean ± standard deviation and median (range) for normally distributed and non-normally distributed data, respectively. The bold values indicate statistical significance at the α = 0.05 level. ^Ψ^ Adjusted *p*-value for multiple testing using Benjamini–Hochberg method. ^£^ Assessed in patients with both stress and rest tests (*n* = 23).

**Table 7:** Per-vessel diagnostic performance in patients followed with invasive coronary angiography (*n* = 16).

	TP	FP	TN	FN	Sensitivity (95% CI)	Specificity (95% CI)	PPV (95% CI)	NPV (95% CI)	Accuracy (95% CI)
Reader 1, SPECT/CT	18	8	14	8	0.69 (0.48–0.86)	0.64 (0.41–0.83)	0.69 (0.48–0.86)	0.64 (0.41–0.83)	0.67 (0.52–0.80)
Reader 1, CZT-SPECT	21	8	14	5	0.81 (0.61–0.93)	0.64 (0.41–0.83)	0.72 (0.53–0.87)	0.74 (0.49–0.91)	0.73 (0.58–0.85)
Reader 2, SPECT/CT	19	12	10	7	0.73 (0.52–0.88)	0.45 (0.24–0.68)	0.61 (0.42–0.78)	0.59 (0.33–0.82)	0.60 (0.45–0.74)
Reader 2, CZT-SPECT	22	11	10	5	0.81 (0.62–0.94)	0.48 (0.26–0.70)	0.67 (0.48–0.82)	0.67 (0.38–0.88)	0.67 (0.52–0.80)

Reference method: invasive coronary angiography, stenosis above 50%. TP, true positive; FP, false positive; TN, true negative; FN, false negative; CI, confidence interval; NPV, negative predictive value; PPV, positive predictive value. **McNemar *p* values are non-significant.**

**Table 8:** Per-patient diagnostic performance in patients followed with invasive coronary angiography (*n* = 16).

	TP	FP	TN	FN	Sensitivity (95% CI)	Specificity (95% CI)	PPV (95% CI)	NPV (95% CI)	Accuracy (95% CI)
**Reader**									
Reader 1, SPECT/CT	12	4	0	0	1.00 (0.73–1.00)	0.00 (0.00–0.60)	0.75 (0.48–0.93)	N/A	0.75 (0.48–0.93)
Reader 1, CZT-SPECT	11	3	1	1	0.92 (0.61–0.99)	0.25 (0.01–0.81)	0.79 (0.49–0.95)	0.50 (0.01–0.99)	0.75 (0.48–0.93)
Reader 2, SPECT/CT	12	4	0	0	1.00 (0.73–1.00)	0.00 (0.00–0.60)	0.75 (0.48–0.93)	N/A	0.75 (0.48–0.93)
Reader 2, CZT-SPECT	12	4	0	0	1.00 (0.73–1.00)	0.00 (0.00–0.60)	0.75 (0.48–0.93)	N/A	0.75 (0.48–0.93)
**Imaging modality**									
SPECT/CT ^£^	6	2	0	5	0.54 (0.23–0.83)	0.00 (0.00–0.84)	0.75 (0.35–0.97)	0.00 (0.00–0.52)	0.46 (0.19–0.75)
CZT-SPECT ^£^	9	1	1	2	0.82 (0.48–0.98)	0.50 (0.12–0.99)	0.90 (0.55–0.99)	0.33 (0.01–0.91)	0.77 (0.46–0.95)

Reference method: invasive coronary angiography, stenosis above 50%. ^£^ Assessed in patients with both stress and rest tests (*n* = 13) with an SDS of three or higher considered positive for CAD. TP, true positive; FP, false positive; TN, true negative; FN, false negative; CI, confidence interval; NPV, negative predictive value; PPV, positive predictive value. **McNemar *p* values are non-significant.**

## Data Availability

The datasets used during the current study are available from the corresponding author on reasonable request.

## References

[1] World Health Organization Cardiovascular Diseases. https://www.who.int/health-topics/cardiovascular-diseases#tab=tab_1.

[2] Tsao C.W., Aday A.W., Almarzooq Z.I., Alonso A., Beaton A.Z., Bittencourt M.S., Boehme A.K., Buxton A.E., Carson A.P., Commodore-Mensah Y. (2022). Heart Disease and Stroke Statistics-2022 Update: A Report from the American Heart Association. Circulation.

[3] Kincl V., Drozdová A., Vašina J., Panovský R., Kamínek M. (2015). Cadmium–zinc–telluride SPECT scanners—New perspectives in nuclear cardiology. Cor Et Vasa.

[4] Driessen R.S., Raijmakers P.G., Danad I., Stuijfzand W.J., Schumacher S.P., Leipsic J.A., Min J.K., Knuuti J., Lammertsma A.A., van Rossum A.C. (2018). Automated SPECT analysis compared with expert visual scoring for the detection of FFR-defined coronary artery disease. Eur. J. Nucl. Med. Mol. Imaging.

[5] Knuuti J., Wijns W., Saraste A., Capodanno D., Barbato E., Funck-Brentano C., Prescott E., Storey R.F., Deaton C., Cuisset T. (2020). 2019 ESC Guidelines for the diagnosis and management of chronic coronary syndromes. Eur. Heart J..

[6] Patel M.R., Peterson E.D., Dai D., Brennan J.M., Redberg R.F., Anderson H.V., Brindis R.G., Douglas P.S. (2010). Low diagnostic yield of elective coronary angiography. N. Engl. J. Med..

[7] Hughes T., Shcherbinin S., Celler A. (2009). A multi-center phantom study comparing image resolution from three state-of-the-art SPECT-CT systems. J. Nucl. Cardiol. Off. Publ. Am. Soc. Nucl. Cardiol..

[8] Farrell C.M., Pinson J.A., Dennett A.M. (2021). CT Attenuation correction and its impact on image quality of myocardial perfusion imaging in coronary artery disease: A systematic review. Asia Ocean. J. Nucl. Med. Biol..

[9] Werner R.A., Thackeray J.T., Diekmann J., Weiberg D., Bauersachs J., Bengel F.M. (2020). The Changing Face of Nuclear Cardiology: Guiding Cardiovascular Care Toward Molecular Medicine. J. Nucl. Med..

[10] Mititelu R., Stanciu S., Mazilu C., Mititelu T., Mititelu L., Gherman A. (2021). Common artefacts in myocardial perfusion imaging. Rom. J. Mil. Med..

[11] Steven B., Anita M. (2006). Artifacts and Pitfalls in Myocardial Perfusion Imaging. J. Nucl. Med. Technol..

[12] Peters A., Kumar J., Patil P.V. (2019). Diagnostic implications of CZT SPECT and impact of CT attenuation correction. J. Nucl. Cardiol..

[13] Ćorović H., Kuburovic J., Salkica N. (2019). Diagnostic accuracy of attenuation correction in perfusion scintigraphy of myocard. Int. J. Med. Rev. Case Rep..

[14] Slomka P.J., Betancur J., Liang J.X., Otaki Y., Hu L.H., Sharir T., Dorbala S., Di Carli M., Fish M.B., Ruddy T.D. (2020). Rationale and design of the REgistry of Fast Myocardial Perfusion Imaging with NExt generation SPECT (REFINE SPECT). J. Nucl. Cardiol..

[15] Niimi T., Nanasato M., Sugimoto M., Maeda H. (2017). Evaluation of Cadmium-Zinc-Telluride Detector-based Single-Photon Emission Computed Tomography for Nuclear Cardiology: A Comparison with Conventional Anger Single-Photon Emission Computed Tomography. Nucl. Med. Mol. Imaging.

[16] Vachatimanont S., Sirisalipoch S., Chantadisai M. (2022). Comparison of the Diagnostic Performance of Myocardial Perfusion Scintigraphy with and without Attenuation Correction. Mol. Imaging Radionucl. Ther..

[17] Benjamini Y., Hochberg Y. (1995). Controlling the False Discovery Rate: A Practical and Powerful Approach to Multiple Testing. J. R. Stat. Soc. Ser. B.

[18] Gambhir S.S., Berman D.S., Ziffer J., Nagler M., Sandler M., Patton J., Hutton B., Sharir T., Haim S.B., Haim S.B. (2009). A novel high-sensitivity rapid-acquisition single-photon cardiac imaging camera. J. Nucl. Med..

[19] Santarelli M.F., Mori A., Bertasi M., Positano V., Gimelli A., Scipioni M., Marzullo P., Landini L. (2021). CZT Detectors-Based SPECT Imaging: How Detector and Collimator Arrangement Can Determine the Overall Performance of the Tomograph. Electronics.

[20] Dondi M., Rodella C., Giubbini R., Camoni L., Karthikeyan G., Vitola J.V., Einstein A.J., Arends B.J., Morozova O., Pascual T.N. (2020). Inter-reader variability of SPECT MPI readings in low- and middle-income countries: Results from the IAEA-MPI Audit Project (I-MAP). J. Nucl. Cardiol..

[21] Angelidis G., Valotassiou V., Tsougos I., Tzavara C., Psimadas D., Theodorou E., Ziaka A., Giannakou S., Ziangas C., Skoularigis J. (2022). Automated Analysis vs. Expert Reading in Nuclear Cardiology: Correlations with the Angiographic Score. Medicina.

[22] Liu C.J., Cheng J.S., Chen Y.C., Huang Y.H., Yen R.F. (2015). A performance comparison of novel cadmium-zinc-telluride camera and conventional SPECT/CT using anthropomorphic torso phantom and water bags to simulate soft tissue and breast attenuation. Ann. Nucl. Med..

[23] Bonnefoy P.B., Janvier L., Arede C., Drouet C., Harami D., Marque S., Ahond-Vionnet R. (2022). Reduced acquisition time for thallium myocardial perfusion imaging with large field cadmium-zinc-telluride SPECT/CT cameras: An equivalence study. J. Nucl. Cardiol..

[24] Gimelli A., Bottai M., Giorgetti A., Genovesi D., Kusch A., Ripoli A., Marzullo P. (2011). Comparison between ultrafast and standard single-photon emission CT in patients with coronary artery disease: A pilot study. Circ. Cardiovasc. Imaging.

[25] Rawal H., Mehta R., Gupta N., Sattiraju S., Mehta S. (2018). Comparison of high efficacy czt spect myocardial perfusion imaging (mpi) and na-i spect mpi with gold standard coronary angiography. J. Am. Coll. Cardiol..

[26] Agostini D., Marie P.-Y., Ben-Haim S., Rouzet F., Songy B., Giordano A., Gimelli A., Hyafil F., Sciagrà R., Bucerius J. (2016). Performance of cardiac cadmium-zinc-telluride gamma camera imaging in coronary artery disease: A review from the cardiovascular committee of the European Association of Nuclear Medicine (EANM). Eur. J. Nucl. Med. Mol. Imaging.

[27] Oddstig J., Martinsson E., Jögi J., Engblom H., Hindorf C. (2019). Differences in attenuation pattern in myocardial SPECT between CZT and conventional gamma cameras. J. Nucl. Cardiol..

[28] Motwani M. (2022). You might be correct, but it makes no difference: No impact of attenuation correction for SPECT MPI on downstream testing. J. Nucl. Cardiol..

